# Pricing Strategies in Dual-Channel Reverse Supply Chains Considering Fairness Concern

**DOI:** 10.3390/ijerph16091657

**Published:** 2019-05-13

**Authors:** Di Wu, Juhong Chen, Ruyu Yan, Ruijun Zhang

**Affiliations:** School of economics and management, Xi’an University of Technology, Xi’an 710048, China; chenjh@xaut.edu.cn (J.C.); yanzi@stu.xaut.edu.cn (R.Y.); zhangruijun01@126.com (R.Z.)

**Keywords:** pricing strategies, dual-channel reverse supply chain, fairness concern, online recycling, Stackelberg game

## Abstract

The fierce competition in the recycling industry and the rapid development of internet technology has prompted recycling centers to develop a dual-channel reverse supply chain with both offline and online recycling channels. After the introduction of online channels, recycling centers and third-party recyclers (TPR) have paid attention to the division of profits in supply chain systems and the behavior of fairness concerns. Therefore, it is necessary to help recycling enterprises make pricing decisions in consideration of fairness concerns. This paper is aimed at answering the following two main questions: (1) When the recycling center or TPR have fairness concerns, how does the optimal pricing and revenue of supply chain members change when both sides are neutral? (2) When the fairness concern coefficient changes, how does the overall revenue of the supply chain system change? How should supply chain members adjust their pricing decisions to maximize their own profits? In order to solve the above problems, Stackelberg game models were made from three aspects: both sides are neutral, only the TPR has fairness concerns, and only the recycling center has fairness concerns. Based on the results of the example analyses for the model, we found that when only the TPR has fairness concerns, the profit of the recycling center and the transfer price of offline channels will decrease, while the profit of TPR is the opposite. Furthermore, when only a recycling center has fairness concerns, it will lead to the reduction of not only the recycling price and transfer price of offline channels, but also the profits of the entire supply chain system. Specially, whether it is for a recycling center or TPR, a lower level of fairness concern coefficient has a stronger impact on pricing and revenue than at high levels.

## 1. Introduction

Over the recent decade, the continuous innovation of electronic technology and consumers’ fanatical pursuit of a new generation of electronic products have not only accelerated the speed of electronic product replacement, but also led to the continuous growth of the number of obsolete and abandoned electronic products [1]. In the four years from 2014 to 2018 alone, the number of waste electrical and electronic equipment (WEEE) in the world increased from 41.8 million tons to 50 million tons, and a “garbage-besieged city” became a trend of globalization [2]. Under such a grim reality, China’s recycling enterprises, while retaining the traditional offline recycling channels with low recycling volume, logistics efficiency, and recycling conversion rates, combine internet technology with garbage recycling and actively develop online recycling channels where websites and mobile phone applications directly contact and trade with consumers. For example, GEM, one of China’s largest recycling centers, has vigorously developed online recycling channels through cooperation with the online platform Loving Recycling on the basis of retaining traditional offline recycling channels. This kind of dual-channel reverse supply chain (DRSC), which coexists between online and offline recycling, has been widely focused on by the recycling industry and academia. However, there are many differences between online and offline recycling channels in DRSC. For example, online channels usually charge higher prices for consumers than offline channels. In addition, online channels use apps to communicate with consumers and express delivery personnel to pick up items at any time, so its convenience has also become the reason why consumers choose it, which have also made offline recyclers feel threatened. Therefore, around the differences and conflicts between different channels in DRSC and other factors, scholars have studied them from the perspectives of pricing decisions, recycling processes, revenue models, contract coordination, and other aspects [3]. Among them, the recovery pricing problem will not only affect the upstream and downstream relationship of the supply chain, consumers’ recovery willingness, and recovery amount, but will also affect the unit operation and maintenance profit of recovery enterprises [4]. Therefore, it is of great significance to set a reasonable recovery price to promote consumers’ recovery amounts, reduce the conflicts between supply chain channels, and enhance the competitiveness of recovery enterprises.

On the other hand, with the increasingly fierce market competition in DRSC, the relationship between supply chain members has become complicated. Therefore, in its economic activities, the traditional assumption of “Rational Economic Man” can no longer accurately describe the economic problems that have occurred [5]. The rational economic man hypothesis refers to the principle of maximizing one’s own interests when making decisions in economic activities [6,7]. However, behavioral economists have found in real cases that the behavior of decision-makers does not fully follow the assumptions of the rational economic man, and even contradicts it [8]. They also point out that when decision-makers have a sense of fairness, the profit level of themselves and other members of the supply chain will have an important impact on their decisions [9]. This is exactly corresponds to the phenomenon that in DRSC, the third party recycler (TPR) of offline channels has its own interests split due to the introduction of online channels, and then adopts retaliatory pricing [10]. Due to the presence of fairness concerns, TPRs also face a series of difficulties, such as being forced to lower the offline recycling price, which hurts the recycling center and reduces its own profits. For example, TPRs need to constantly bargain with the recycling center, and since the recycling center occupies a dominant position in DRSC, this process is difficult and lasting. In the face of these difficulties, TPRs should communicate its own views and possible solutions with recycling centers in a timely manner, in order to make all members and systems in the supply chain upgrade. In the existing research on supply chain management, the influence of different supply chain structures, supply chain members, and different fairness concerns on pricing decisions and profit acquisition of members has been deeply studied in academia [11,12,13]. However, due to the short time of DRSCs’ appearance and the immaturity of theoretical research, few scholars have conducted any research on DRSC pricing that takes into account fairness concerns. 

Literature has extensively examined pricing of the reverse supply chain. Guide [14] pointed out that remanufacturers can control the demand, quantity, and quality of products through recycling price decisions. Debo [15] discussed the pricing of the market composed of heterogeneous consumers and pointed out that reducing the high cost of disposable products is the key driver to promote recycling and remanufacturing. Gu [16] studied the reverse supply chain system composed of a single manufacturer and a single retailer from the perspectives of non-cooperative games and cooperative games, respectively, pointing out that the system profit is lower in a non-cooperative equilibrium than that in a cooperative equilibrium, and discussed its coordination strategy. On the basis of Gu’s research, Wang [17] used Stackelberg game theory to study the pricing strategy of reverse supply chains under a multi-retailer structure, and improved the profit of the whole supply chain by making a joint pricing strategy. In addition, some scholars have conducted further research on reverse supply chains from default risk [18], incentives for environmental research [19], and optimization of demand [20] in recent years. However, the above research on the pricing of reverse supply chains were conducted before online recycling existed. In order to analyze the influencing factors of pricing problems in DRSC more comprehensively, the related literature is integrated in this paper.

Until now, scholars have studied DRSC’s recovery pricing from the aspects of consumer preferences, channel power, regional differences, government subsidies, and stability. Feng [21] discussed the pricing strategies under the three modes of single-line offline recycling channels, single-line recycling channels, and double-line recycling channels under the guidance of the recycling center, pointed out that double-line recycling channels can maximize the revenue of the recycling center and the whole system, and construct a revenue sharing contract for double-line recycling channels around consumer preferences to coordinate supply chain partnerships. Giri [22] analyzed DRSC pricing and returned decisions under five different channel powers: centralized, decentralized, manufacturer-led, retailer-led, and third-party-led. Through numerical analysis, he pointed out that retailer-led schemes can bring higher returns to the supply chain. Chen and Wu [3] further studied how TPR in different regions can make decisions on three pricing strategies of keeping prices unchanged, unifying all prices, and maximizing their own profits when introducing online channels to the recycling centers in DRSC. Ma [23] established Stackelberg game models of online recyclers, offline recyclers, and remanufacturers under the government subsidy mode based on customer utility expectations. They pointed out that although the improvement of service quality and the reduction of logistics cost will lead to the reduction of system stability, it can improve the revenue of the supply chain system, and the system stability through government coordination. Although the above research provides certain theoretical basis for a DRSC’s pricing decision, it is all based on the assumption of a rational person. On the other hand, the issue of supply chain pricing, which considers fairness concerns, has been extensively studied by academia. 

As mentioned before, many researchers have found that the fairness concerns of supply chain members will affect their pricing decisions. At first Ho [24] confirmed the existence of fairness concerns behavior in the supply chain through experiments, and pointed out that the distribution of benefits among supply chain entities is the reason for the existence of fair preference. Later Bolton and Rabin [25] quantified the decrease in profits of supply chain enterprises caused by fairness concerns using functional expressions respectively. Cui [26] introduced the concept of fairness concerns into the study of pricing and coordination of dual-channel supply chains, and pointed out through numerical simulation that the fairness concerns of supply chain members can be met by increasing wholesale prices, thus realizing the coordination of supply chain systems and maximizing profits. Caliskan [27], based on Cui’s research, extended the linear function demand in the revenue model to the exponential functions, and pointed out that the exponential demand function is easier to use to realize the coordination of the supply chain. Katok [28] considered fairness concerns and bounded rationality in his supply chain decision-making model and proved that when the participants’ fairness concerns belong to private information, the supply chain cannot be coordinated. However, few studies researched the impact of fairness concern behavior on pricing decisions and revenue maximization of enterprises in DRSC. 

Motivated by the above facts, the paper explores the pricing problem of DRSC considering fairness concerns. In a DRSC’s offline channel, consumers recycle WEEE to a TPR and then the TPR transfers it to the recycling center. In a DRSC’s online channel, consumers directly recycle WEEE to the recycling center. Therefore, the recycling center and TPR not have only the cooperation relationship of an offline channel, but also the competition relationship of online channel. In their Stackelberg game, recycling centers dominate. At the same time, we respectively consider the optimal pricing strategy and the maximum profit under the three circumstances that both parties are neutral, only the recycling center has fairness concerns, only TPR has fairness concerns, and compare them. 

More specifically, we address the following research questions: 

(1) When the recycling center or TPR have fairness concerns, respectively, how does the optimal pricing and revenue of supply chain members in DRSC change when both are neutral? 

(2) When the fairness concern coefficient of the recycling center or TPR changes, how does the overall revenue of DRSC system change? How should supply chain members adjust their pricing decisions to maximize their own profits? 

Through analysis of the model and data results, we have made some meaningful findings different from previous studies. First of all, our research background is different from most previous studies. Although there has been a small amount of research on DRSC, the mainstream research background for supply chain in academia is still a dual-channel supply chain with positive channels or a reverse supply chain with multiple offline channels. As a combination of the Internet, recycling, environmental protection, policy guidance, and other academic hot topics, the research on DRSC will surely become one of the most popular issues in the academic community in the future. It is also because of this that the exploration of such problems at this stage is of great significance. Secondly, our research subjects and considerations are different from previous studies. Although all the research is aimed at the pricing decision-making of recycling enterprises, unlike the previous research on DRSC, which all regard recycling enterprises as “rational economic persons”, our research takes into account the irrational behavior factors of decision-makers of recycling enterprises. Through the numerical analysis of the fairness concern coefficient of decision-makers from low to high, we have explored its influence on the pricing and revenue changes of recycling enterprises in DRSC and its deeper management significance. Finally, our research conclusion is different from previous studies. After numerical analysis of the behavioral factors of recycling enterprises in DRSC, we found some interesting conclusions different from previous studies. For example, we found that when the recycling center has fairness concerns, it will not only damage the profits of a TPR and the supply chain system, but also reduce the profits of the recycling center itself, resulting in the phenomenon of “harm others and harm oneself”. In addition, we also find that when the fairness concern coefficient of one supply chain member increases, its influence on the pricing and profits of the other member gradually weakens. Furthermore, our study is also of great significance for maintaining the coordination of the recycling industry, improving the profits of recycling enterprises and further promoting the sustainable development of the ecological environment.

The main contribution of the paper is threefold. First, our research is one of the few studies on DRSC based on online recycling, which studies the impact of fairness concerns of its supply chain members on the optimal pricing strategy of recycling enterprises. Second, through the construction and solution of the Stackelberg game model and the analysis of examples, we have verified the law that when different supply chain members have fairness concern behavior, the profits of enterprises and supply chain systems change with the increase of their fairness concern coefficient. Third, we have provided specific management suggestions to the recycling center and the TPR under different circumstances, including adjusting pricing strategies and controlling fair behavior. 

This paper is organized as follows. Section 2 introduces the notations and assumptions for the recycling center and TPR in the models. In Section 3, we respectively construct revenue models of the recycling center and TPR in three scenarios, and solve and prove the models. Section 4 conducts a numerical example to discuss the impact of fairness concerns on pricing and revenue of supply chain members in different situations based on the analysis of data results. In Section 5, we provide concluding remarks.

## 2. Model Assumptions and Notations 

The goal of this paper is to solve the pricing strategy problem of the recycling center and TPR in a DRSC considering fairness concerns. Therefore, on the one hand, we established a DRSC mathematical model based on a Stackelberg game under neutrality. In the offline channel of this model, consumers first recycle WEEE to a TPR at a certain offline recycling price, then the TPR resells it to the recycling center at a transfer price, and the TPR earns the difference. However, consumers who choose online channels will directly recycle WEEE to the recycling center through express delivery and door-to-door pick-up, etc. After obtaining WEEE, the recycling center will disassemble, decompose, and send WEEE to chemical plants or remanufacturers. We assume that the recycling center can obtain the same income from the treatment of unit WEEE. On the other hand, by introducing the fairness concerns of the recycling center and TPR into the above models, we respectively constructed two pricing models, namely, the recycling center has fairness concerns and TPR has fairness concerns. In these two models, supply chain members not only pay attention to their own income, but also observe the income of other members based on their own income. When they think they have suffered injustice, they will take action (even sacrifice their own interests) in exchange for a sense of fairness. Based on Chen’s [3] research, we put forward the following assumptions for the construction and solution of the following model from the aspects of the recovery amount, channel power, information balance, etc. 

**Assumption 1.** The channel power of the recycling center in the supply chain is far greater than the TPR, so it occupies the dominant position in their Stackelberg game. Not only does it have strong economic strength, but the recycling center is also supported by the government in terms of policies because it is more environmentally friendly. At the same time, the TPR is usually a small recycling enterprise, which is not only lacking in financial strength, but also lacks a means of decomposing and disposing of WEEE. All they can do is resell the recycled WEEE to the recycling center at a higher price. Therefore, the recycling center is the dominant player in a DRSC. 

**Assumption 2.** The information obtained by each member of the supply chain is balanced, regardless of the supply chain inefficiency, risk sharing, and bullwhip effect of demand and supply caused by information asymmetry and differences in information utilization capabilities. Every enterprise has no hidden information. At the same time, the variables on which decisions are based and the state of the variables are transparent and known to all other enterprises in the supply chain. 

**Assumption 3.** We are studying the same type of WEEE with the same degree of loss and recovery conversion. Although online recycling channels can recycle WEEE such as mobile phones, digital cameras, notebook computers, televisions, and other types, and there is no WEEE with absolutely the same degree of loss in reality, these are not the issues that this study focuses on. Therefore, in order to simplify the study, we make this assumption. 

**Assumption 4.** We assume that the recovery of online and offline is linearly related to its respective recovery price, and the recovery amount will increase with the increase of the recovery price. In addition, the recovery amount is also affected by consumers’ preferences for different channels. That is, if *θ* (0 < *θ* < 1) represents the proportion of consumers who prefer online channels, then the proportion of consumers who prefer offline channels is 1–*θ*. Based on this, we assume that the recovery amount of the offline channel in a DRSC is *d_r_* = (1 − *θ*)*a* + *βp_t_* − *κp_e_*, and the recovery amount of the online channel is *d_e_* = *θa* + *βp_e_* − *κp_t_*. 

**Assumption 5.** In this study, fairness utility functions *μ_m_* and *μ_t_*, as well as fairness concerns *λ* and *η*(*λ*, *η* > 0) are introduced for recycling centers and TPRs, respectively. Based on Kahneman’s [29] point of view, we assume that when supply chain members have fairness concerns, their fair utility is related to their fairness concerns, their own benefits, and the benefits of other supply chain members. 

The notations in this paper are shown in Table 1. 

## 3. Model Solution

**Figure 1:**
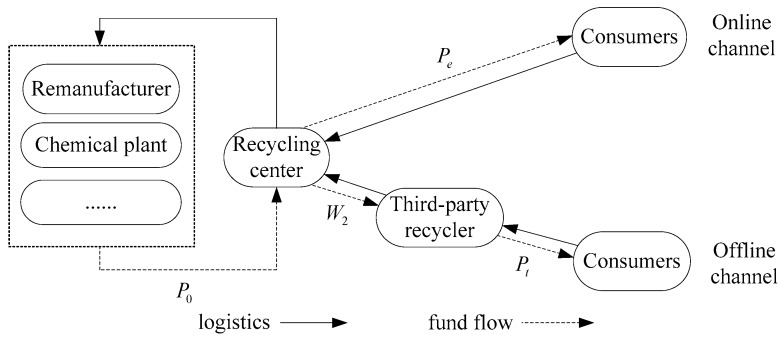
Depiction of the dual-channel reverse supply chain (DRSC).

In this section, we will study the pricing strategies of a DRSC from three parts: all supply chain members are neutral, only recycling centers have fairness concerns, and only the TPR has fairness concerns. In offline channels, consumers sell WEEE to TPRs at a unit price *p_t_*, and the TPR transfers WEEE to recycling centers at unit price *w* through collection, sorting, and classification. In online recycling channels, consumers sell WEEE directly to recycling centers at unit price *p_e_*. The recycling center classifies WEEE recovered in online and offline channels and sells them to upstream remanufacturing enterprises or factories for further detection, decomposition, and centralized treatment at unit price *p*_0_. In this mode, the recycling center and TPR have both vertical cooperation and horizontal competition, so the situation of the supply chain is more complex. The supply chain structure diagram of a DRSC is shown in Figure 1. 

As the basis of later research, we first describe the profit function of the recycling center and TPR in a DRSC. According to the assumptions in Section 2, the following can be obtained: 

The revenue of the recycling center is ∏*_m_* = (*p*_0_ − *w*)((1 − *θ)a* + *βp_t_* − *κp_e_*) + (*p*_0_ − *p_e_* − *c*)(*θa* + *βp_e_* − *κp_t_*), TPR’s revenue is ∏*_t_* = (*w* − *p*_t_)((1 − *θ*)*a* + *βp_t_* − *κp_e_*).

On the one hand, the revenue ∏*_m_* model formulation of the recycling center is mainly composed of online and offline revenue. Its income is obtained by multiplying the unit profit of online or offline channels and the recovery amount. Among them, online and offline recovery is affected by basic recovery, consumer preferences for different channels, and recycling price. Unit profit of online channels is affected by unit disposal price, online recycling price, and logistics cost. The unit profit of offline channels is determined by the unit disposal price and offline transfer price. On the other hand, TPR’s revenue ∏*_t_* is obtained by multiplying the recovery amounts of offline channels and the unit profit, and its unit profit is affected by offline recycling price and transfer price.

In order to simplify the calculation, on the premise of not interfering with our research on the impact of the fairness concern coefficient on pricing strategy, we assigned elasticity coefficients *β* and *κ* to the values of 5 and 3, respectively, for price effects on recovery. Therefore, the revenue of the recycling center and TPR can be expressed as follows:

(1) $$\prod_{m}=(p_{o}-w)(\alpha -\theta \alpha +5p_{t}-3p_{e})+(p_{o}-p_{e}-c)(\theta \alpha +5p_{e}-3p_{t})$$

(2) $$\prod_{t}=(w-p_{t})(\alpha -\theta \alpha +5p_{t}-3p_{e})$$

### 3.1. Both Sides Are Neutral

In this case, the recycling center and TPR are both neutral. That is, they only make pricing decisions according to their own income, and do not pay attention to the income of other members of the supply chain. Due to the dominant position of the recycling center in the Stackelberg game with a TPR, the recycling center makes pricing decisions for *p_e_* and *w* first. As a follower of the recycling center, after observing the recycling center’s decision, the TPR makes the best decision for *p_t_*. According to backward induction to solve this model, the follower’s decision should be calculated first.

First, we solve the first and second partial derivatives of ∏_*t*_ to *p_t_* according to Equation (2) and get ∂^2^∏_*t*_/∂*p_t_^2^* = −10 < 0. Therefore, it can be seen that ∏_*t*_ is concave, that is, there is an optimal p_t_^*^, so that ∏_*t*_ can obtain a maximum value. So, let ∂∏_*t*_/∂*p_t_* = 0, and get p_t_^*^ as follows:

(3) $${p_{t}}^{*}=\left[5w+3p_{e}+\alpha (\theta -1)\right]/10$$

Next, we solve the first partial derivatives of ∏_*m*_ with respect to *p_e_* and *w* according to Equation (1), and the results are as follows:

(4) ∂Πm∂pe=30w−41c+26po−3α−7αθ−82pe

(5) ∂Πm∂w=3c+2po+6pe−α+αθ−10w

**Property** **1.** *The objective function ∏_m_(p_e_, w) is always concave with p_e_ and w.*

Property 1 (proofs can be found in Appendix A) indicates that we can obtain the optimal online recycling price p_e_^*^ and offline transfer price w^*^ of the recovery center by setting Equations (4) and (5) to 0 and combining the results:

(6) $$p_{e}^{*}=\frac{16p_{o}-16c-3\alpha -2\alpha \theta }{32}$$

(7) $$w^{*}=\frac{80p_{o}-25\alpha +10\alpha \theta }{160}$$

Substituting Equations (6) and (7) into (5), the final optimal TPR recovery price p_t_^**^ is obtained:

(8) $$p_{t}^{**}=\frac{64p_{o}-24c-33\alpha +18\alpha \theta }{160}$$

### 3.2. Only the TPR Has Fairness Concerns 

In this mode, the recycling center is neutral, while the TPR has fairness concerns. That is, the TPR will not only pay attention to its own income, but will also compare the income of the recycling center with its own. The TPR will adjust the pricing strategy when it finds that the revenue of the recycling center is too high and considers itself as “weak” in the supply chain to counter unfairness and jealousy; even if it will harm its own income, it must also obtain its own sense of fairness. Therefore, we introduce the fairness concern coefficient *λ* (*λ* > 0) of the TPR and obtain the fair utility function *μ_t_* of the TPR. In the formulation for *μ_t_*, we use Bolton’s [25] assumption for reference. He pointed out that enterprises are not only motivated by absolute income, but also tend to be influenced by relative income. Therefore, when describing the utility function, he assumed that the difference in profits will bring about a change in utility. When one’s own profit is higher than others, one’s own utility increases; when lower, one’s utility decreases. Specifically, we assume that the fair utility of the TPR is affected by the TPR’s income ∏_*t*_, fairness concern coefficient *λ* and its difference with the recycling center’s income. Therefore, we introduce the fairness concern coefficient *λ* (*λ* > 0) of the TPR and obtain the fair utility function *μ_t_* of the TPR at this time, as follows:

(9) $$\mu_{t}=\prod_{t}-\lambda (\prod_{m}-\prod_{t})=(1+\lambda )\prod_{t}-\lambda \prod_{m}$$

The profit functions of the recovery center and the TPR in Equations (1) and (2) are substituted into Equation (9), and the first and second partial derivatives of *μ_t_* to *p_t_* are solved to obtain ∂^2^*μ_t_*/∂*p_t_^2^* = −10(1 + λ). Because when *λ* > 0, there are ∂^2^*μ_t_*/∂*p_t_^2^* < 0. Therefore, it can be seen that *μ_t_* is concave, that is, there is an optimal p_t_^*^, which makes *μ_t_* obtain a maximum value. Let ∂*μ_t_*/∂*p_t_* = 0, and the solution of p_t_^*^ is:

(10) $${p_{t}}^{*}=\frac{5w+3p_{e}-\alpha +\alpha \theta +10\lambda w-3c\lambda -2\lambda p_{o}-\alpha \lambda +\alpha \theta \lambda }{10+10\lambda }$$

Next, we substitute p_t_^*^ into the profit function ∏_*m*_ of the recovery center in Formula (1), and obtain the first order partial derivatives ∂∏_*m*_/∂*p_e_*, ∂∏_*m*_/∂*w* of ∏_*m*_ to *p_e_* and *w*, respectively, and obtain the following solutions:

(11) ∂Πm∂pe=26po−3α−7αθ+14λpo−3αλ−7αθλ+30b(1+2λ)−c(41+59λ)−(82+100λ)pe

(12) ∂Πm∂w=3c+2po+6pe−α+αθ+9cλ+6λpo+12λpe−αλ+αθλ−(10+20λ)w

**Property** **2.** *The objective function ∏_m_(p_e_, w) is always concave with p_e_ and w.*

Property 2 (proofs can be found in Appendix A) indicates that we can obtain the optimal online recovery price p_e_^*^ and offline transfer price w^*^ of the recovery center by setting Equations (11) and (12) to 0, respectively, and then combining them:

(13) $$\begin{array}{l} w^{*}=\frac{3c+2p_{o}-\alpha +\alpha \theta +9c\lambda +6\lambda p_{o}-\alpha \lambda +\alpha \lambda \theta }{10+20\lambda }\frac{82+100\lambda }{82+100\lambda -3(1+\lambda )\left(12\lambda +6\right)/\left(1+2\lambda \right)}\\ +(\frac{12\lambda +6}{10+20\lambda })(\frac{26p_{o}-3\alpha -4\alpha \theta +14\lambda p_{o}-7\alpha \theta \lambda -c(41+59\lambda )}{82+100\lambda -3(1+\lambda )\left(12\lambda +6\right)/\left(1+2\lambda \right)}) \end{array}$$

(14) $$p_{e}^{*}=\frac{16p_{o}-16c-3\alpha -2\alpha \theta }{32}$$

Substituting Equations (13) and (14) into Equation (10), the final optimal recovery price p_t_^**^ of the TPR is obtained as follows:

(15) $$p_{t}^{**}=\frac{64p_{o}-24c-33\alpha +18\alpha \theta }{160}$$

### 3.3. Only the Recycling Center Has Fairness Concerns

In this mode, the TPR is neutral, and recycling centers have fairness concerns. At this time, the TPR’s decision will be based on the maximum of its own income to make the most favorable pricing decision, while the recycling center will compare its own and the TPR’s income due to its fairness concerns. Despite being the dominant player in a DRSC and the profit being definitely higher than a TPR’s, the recycling center will adjust its pricing strategy if it feels that the profit obtained by a TPR is higher than the fair situation it expects. Based on this, we introduce the fairness concern coefficient *η* (*η* > 0) of the recycling center, and obtain the fair utility function *μ_m_* of the recycling center. Similar to the characterization of the fairness concern utility in Section 3.2, we describe *μ_m_* at this time as follows:

(16) $$\mu_{m}=\prod_{m}-\eta (\prod_{t}-\prod_{m})=(1+\eta )\prod_{m}-\eta \prod_{t}$$

Since a TPR is a follower in a DRSC, it must still calculate its optimal decision. Because ∏_*t*_ has been proved to be concave in Section 3.1, there is an optimal p_t_^*^ that makes ∏_*t*_ obtain the maximum value. So let ∂∏_*t*_/∂*p_t_* = 0, and get p_t_^*^ as follows:

(17) $${p_{t}}^{*}=\left[5w+3p_{e}+\alpha (\theta -1)\right]/10$$

Next, we will solve the decision of the recycling center as the leader. The profit functions of the recovery center and TPR in Equations (1) and (2) are substituted into Equation (16), and the first partial derivatives ∂*μ_m_*/∂*p_e_*, ∂*μ_m_*/∂*w*, and the second partial derivatives ∂^2^*μ_m_*/∂*p_e_*^2^, ∂^2^*μ_m_*/∂*w*^2^, of *w* and *p_t_* are solved for *μ_m_*.

**Property** **3.** *The objective function μ_m_(p_e_, w) is always concave with p_e_ and w.*

Property 3 (proofs can be found in Appendix A) indicates that the recycling center has the optimal online recycling price p_e_^*^ and offline transfer price w^*^, which makes the utility of the recycling center reach the maximum value. Therefore, we make ∂*μ_m_*/∂*p_e_* = 0, ∂*μ_m_*/∂*w* = 0, respectively, and carry out simultaneous equations to obtain the final solution:

(18) $${p_{e}}^{*}=\frac{-16c+16p_{o}-3\alpha -2\alpha \theta }{32}$$

(19) $$w^{*}=-\frac{-160p_{o}+50\alpha -52\alpha \theta +48\eta c-208\eta p_{o}+91\alpha \eta -78\alpha \theta \eta +32\alpha \theta +32\alpha \eta \theta }{160(2+3\eta )}$$

Substituting Equations (18) and (19) into Equation (17), the final optimal recovery price p_t_^**^ of the TPR is obtained as follows:

(20) $${p_{t}}^{**}=-\frac{48c-128p_{o}+66\alpha -20\alpha \theta +96\eta c-176\eta p_{o}+107\alpha \eta -30\alpha \theta \eta -16\alpha \theta +32\alpha \eta \theta }{160(2+3\eta )}$$

By substituting p_e_^*^, w^*^, p_t_^**^ in Section 3.1, Section 3.2, and Section 3.3, respectively, into Equations (1) and (2), the revenue ∏_*m*_ of the recycling center, the revenue ∏_*t*_ of the TPR, and the total revenue ∏_*m*_ + ∏_t_ of the supply chain system can be obtained under the corresponding situation.

## 4. Example Analysis

In this section, we present numerical examples to verify the results of the models in Section 3.2 and Section 3.3. By assuming the relevant parameters, we will verify numerically the impact of fairness concerns of the recycling center and TPR in a DRSC on the pricing and revenue of supply chain members, and explore the causes and future countermeasures based on the analysis of data results and trends. The values of various model parameters are listed as follows: *p*_0_ = 200, *α* = 100, *c* = 40, *θ* = 0.4. 

### 4.1. The TPR Has Fairness Concerns 

**Figure 2:**
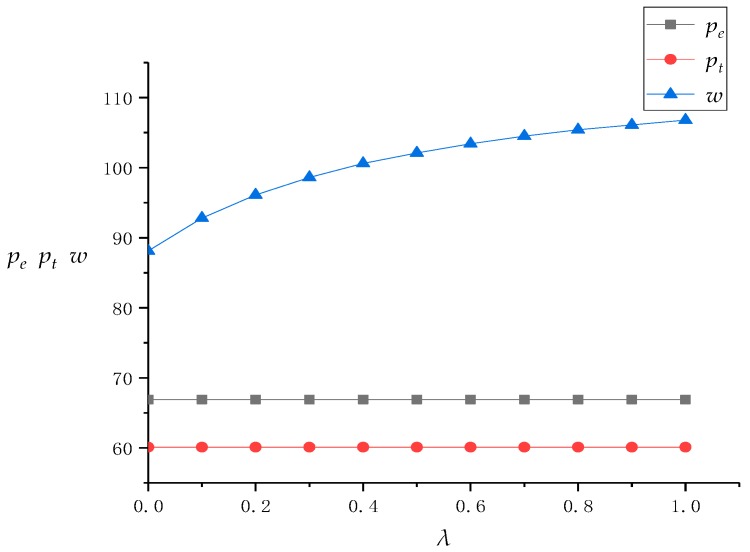
The change of prices as λ increases.

**Figure 3:**
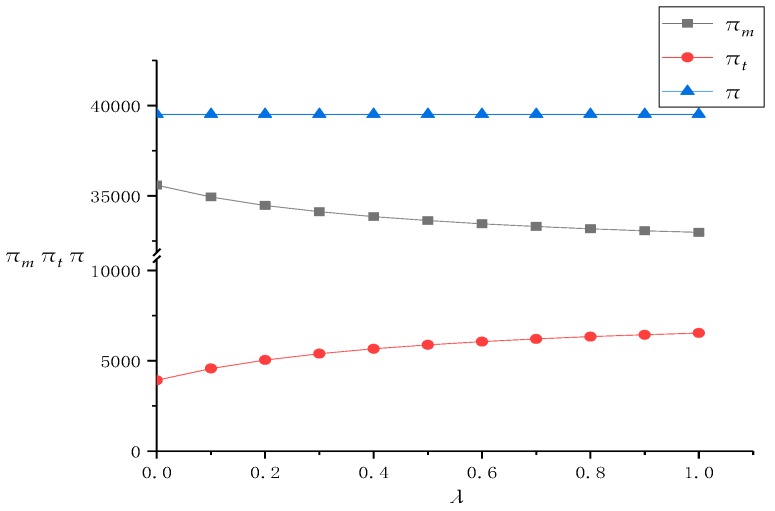
The change of profits as λ increases.

First of all, we conducted a numerical analysis on the solution results of the model in Section 3.2. In the DRSC mode of this situation, the recycling center does not have fairness concerns. Because the recycling center introduces online channels to divide the recycling market and TPR revenue, the fairness concern coefficient *λ* of the TPR gradually increases from 0 to 1. Based on this, we use Mathematica software to obtain the optimal pricing of the recycling center, TPR, and the revenue value of the entire supply chain system, as shown in Table 2. At the same time, we also draw a line chart of the influence of *λ* on the pricing and revenue of supply chain members in Figure 2 and Figure 3 to further analyze it. 

Figure 2 and Figure 3 first show that with the promotion of *λ*, the transfer price *w* for the TPR of the recycling center in an offline channel gradually increases, the profit of the recycling center gradually decreases, the profit of the TPR gradually increases, and the profit difference between the recycling center and the TPR continuously decreases. This is because TPR’s bargaining power against recycling centers will continue to increase as the TPR’s fairness concerns continue to rise, forcing recycling centers to raise the transfer price of WEEE and make profit concessions. Therefore, the TPR has also brought about redistribution of supply chain profits when gaining a relatively large voice in the supply chain, and finally improved its own channel profits. At the same time, because the recycling center has raised the transfer price and increased the channel cost while not raising revenue through other channels, its profits are also continuously decreasing. Therefore, this directly leads to the decrease of profit difference between the recycling center and TPR. Such “harming others to benefit oneself” of the TPR has achieved its fairness concerns and shortened the profit gap between itself and the recycling center. The recycling center has reduced its own profits by selling them, but it has also maintained the coordination of the supply chain and put an end to potential unstable factors. 

In addition, although *λ* is constantly changing, the total profits of the supply chain system remain unchanged in homeostasis. This is because although the TPR’s fairness concerns have affected its formulation of the transfer price *w* with the recycling center, the TPR’s bargaining power does not involve the online recycling price of the recycling center, so the formulation of *p_e_* keeps unchanging with *λ* and is not affected by it. The same reason can also explain that the TPR has not changed the offline recovery price *p_t_* for consumers. Therefore, for the whole supply chain system composed of the recycling center and TPR, its income and expenditure have not changed, which leads to the total revenue of the whole supply chain system not being changed, too. 

Finally, with the promotion of *λ*, the rate of profit reduction in the recycling center is continuously decreasing, and the rate of profit increase in the TPR is also continuously decreasing. This is because the increase of transfer price *w* is affected by the fairness coefficient *λ* and is non-linear, that is, although *w* increases with the increase of *λ*, its rate of increase is also decreasing. This is because when the fairness concern coefficient of the TPR is at a low level, the recycling center will pay more attention to its changes, and will raise the transfer price for every little increase in *λ*. This has led to a substantial decrease in its own revenue and a substantial increase in the TPR’s revenue. However, when the fairness concern coefficient of the TPR is at a higher level, the impact of unit changes in *λ* when the recycling center is somewhat weaker than that at a lower level. This has also led to a slower rate of revenue reduction in recycling centers and a slower rate of revenue increase in the TPR. Therefore, this phenomenon shows that although the TPR can improve its profit by improving its fairness concern coefficient and bargaining power, if it pays too much attention to fairness, the increase in profit will be very limited. At the same time, for the recycling center, moderate attention to the fairness concerns of supply chain members and concession of their own profits can promote the stability of supply chain coordination, but if the other party pays too much attention to fairness, the recycling center should not blindly sell its own profits. 

### 4.2. Recycling Centers Has Fairness Concerns

**Figure 4:**
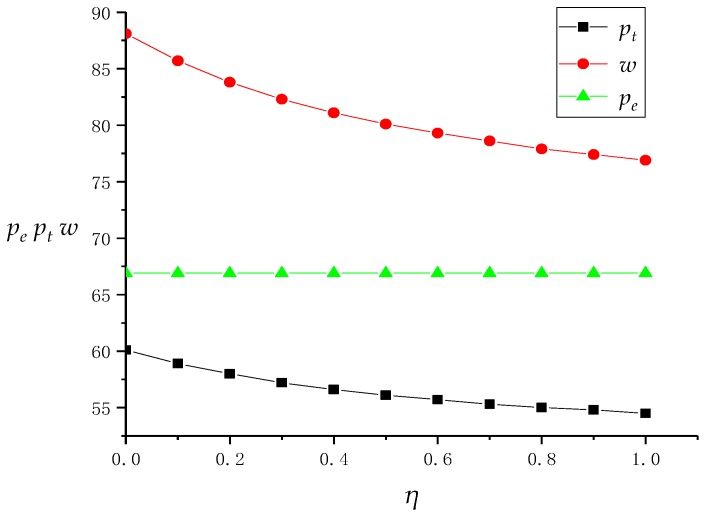
The change of prices as *η* increases.

**Figure 5:**
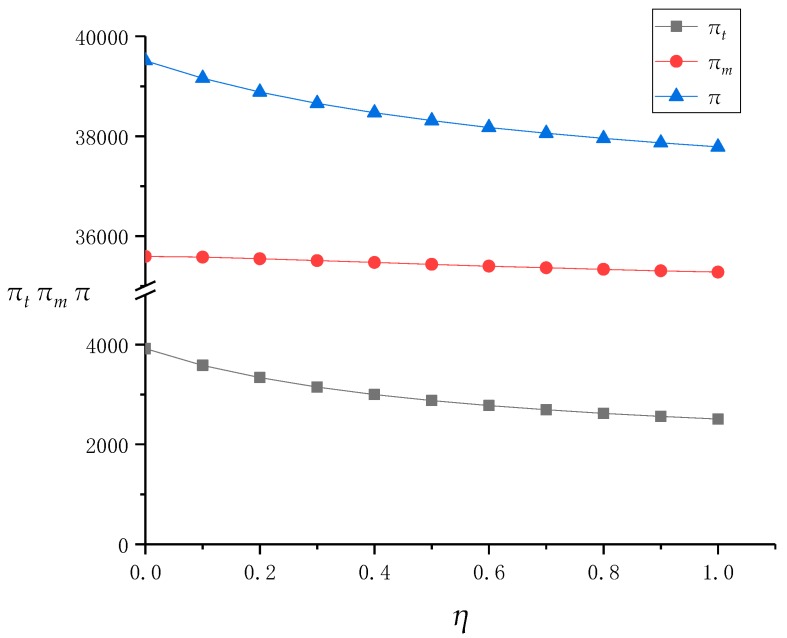
The change of profits as *η* increases.

In this subsection, we analyze the model results in Section 3.3. In the context of the Section 3.3 model, on the one hand, the TPR does not have fairness concerns. On the other hand, although the recovery efficiency and conversion rate of traditional offline recovery channels are low, the market share is still high due to its long existence in the market, which adds difficulties and obstacles to the introduction of online recovery channels based on the Internet for the recycling center. Therefore, the recycling center believes that the profit distribution in a DRSC is unfair, and there is a fairness concern coefficient *η* that gradually increases from 0 to 1. Based on this, similar to the simulation in Section 4.1, we used Mathematica software to obtain the income values of *p_t_*, *w*, *p_e_*, and the entire supply chain system, as shown in Table 3, and their line charts are affected by *η*, as shown in Figure 4 and Figure 5. 

First, figures show that with the increase of *η*, the online recovery price *pe* remains unchanged, and the transfer price *w* and the recovery price *p_t_* in the offline channel gradually decrease, while the profits of the recovery center and TPR are also continuously decreasing. This is because under the influence of fairness concerns, the recycling center will not change the recycling price of its online channels, but will keep down the transfer price of offline channels against the TPR by means of channel power and bargaining strategies. Facing the reduction of transfer price, the TPR has to keep the recovery price down in the process of recovering WEEE from consumers in order to reduce losses as much as possible. 

In addition, the reduction of the TPR’s recycling price directly affects the recovery volume of offline channels, and eventually leads to the reduction of the TPR’s profit. Interestingly, although the recycling center is concerned about fairness in order to better develop online channels, and the increase in *η* does increase the proportion of online channel recycling, in fact, the profit of the recycling center also decreases due to the decrease in offline channel revenue. As the profits of the recycling center and TPR both decrease, the total profits of the supply chain system also decrease. Therefore, although the fairness concerns of the recycling center have widened the profit gap between itself and the TPR and satisfied its own fairness psychology, in fact, the profits of the TPR and the supply chain system have been reduced as well. This behavior of the recycling center can be said to be “damaging others and hurting oneself”. 

Finally, with the increase of *η*, the profit reduction rate of the recycling center, TPR, and supply chain system is continuously decreasing. This is because when *η* rises to a higher stage, its influence on the recycling price and transfer price of offline channels is continuously weakened, and the influence on above profits is also reduced. At this time, even if the recycling center raises its fairness concern coefficient, the profit difference between the TPR and recycling center has stabilized. Therefore, for the recycling center, when *η* is at a lower value, its promotion can significantly widen the profit gap between itself and the TPR. However, if the recycling center pays too much attention to fairness, then when *η* is at a higher value, on the one hand, raising the fairness concerns have almost failed to widen the profit gap with a TPR, on the other hand, it also causes huge losses to a TPR, thus easily causing conflicts between channels. 

### 4.3. Discussion

In this subsection, we summarize and discuss the results of example analyses in Section 4.1 and Section 4.2, respectively. First of all, the numerical results of Section 4.1 show that a TPR’s fairness concern behavior will result in “harming others to benefit oneself”. The TPR’s concerns for fairness led to its dissatisfaction with the distribution of profits in the supply chain and bargaining with the recycling center, which eventually raise the transfer price of the recycling center’s offline channels and significantly increase the TPR’s profits. At the same time, as the profit of the offline recycling channel of the recycling center is divided, its total profit also decreases significantly. In addition, since the TPR and the recycling center only limit the adjustment of pricing strategy to the transfer price of offline channels in this process and affect the recycling price of online and offline channels for consumers, the overall profit of the supply chain system is not changed. The TPR’s fairness concerns increase its profits, reduce the profits of the recycling center, and successfully narrow the profit gap between the TPR and the recycling center. This phenomenon can be explained by the proverb “to enrich oneself at another’s expense”. 

Secondly, the numerical results of Section 4.2 show that the fairness concerns of the recycling center will lead to the result of “damaging others and hurting oneself”. As the lead player in the supply chain, the recycling center is also dissatisfied with the distribution of profits in the supply chain, and will use channel power and bargaining power to meet its fairness concerns. This behavior reduces the transfer price of offline channels, and prompts the TPR to reduce the recycling price of offline channels, resulting in the profits of the recycling center, TPR, and supply chain system being reduced at the same time. Therefore, even if the fairness concerns of the recycling center has successfully widened the profit gap between itself and the TPR, this phenomenon can only be described by the proverb “harm others and harm oneself”. 

Finally, by comprehensively comparing and observing Section 4.1 and Section 4.2, we found that with the increase of the fairness concern coefficient, its influence on profit margins was weakened continuously. Although an enterprise’s fairness concern behavior will initially enhance its bargaining power and prompt other enterprises in the supply chain to make profit concessions, once its fairness concern behavior reaches a higher stage and continues to grow, the increase in its own profit will be very limited. At the same time, the higher level of fairness concerns will greatly reduce the profits of other enterprises, and lead to conflicts, reduce the profits of the supply chain system, and other serious negative consequences. Therefore, enterprises should reasonably understand and control their fairness concern behavior. While paying attention to their own profits, they also need to pay more attention to the systematic reactions of other supply chain members.

## 5. Conclusions

### 5.1. Conclusions and Managerial Implications

This paper investigated the pricing strategies of a dual-channel reverse supply chain with fairness concerns. By aiming at a dual-channel reverse supply chain composed of a recycling center and TPR, a revenue-price model was constructed and solved from three situations: both sides are neutral, only the TPR has fairness concerns, and only the recycling center has fairness concerns. In addition, we conducted a case study on the situation that recycling enterprises have equity concerns. Based on the results of the case study, we discussed the trends of the impact of changes in equity concerns on pricing and profits of enterprises and the supply chain system and the management implications behind it. The main findings are summarized as follows: 

(1) When the TPR perceives the uneven distribution of profits and generates fairness concerns, the increase of its fairness preference will prompt the TPR to adjust its pricing strategy with the recycling center and lead to the increase of its own income and the transfer price of offline channels. Due to the concession of profits, the profits of the recycling center decrease. So far, the TPR has achieved its goal of fairness concerns, shortened the profit difference between the TPR and the recycling center, and led “to enrich oneself at another’s expense”. In addition, the TPR’s behavior only affects the decision-making of the members of the offline channel in the system, so it does not affect the online recycling price, offline recycling price, and the total revenue of the supply chain system for consumers. The TPR’s behavior can be seen as successfully satisfying its sense of fairness and improving its profits. However, if the profit of the recycling center divided by the TPR does not reach a high level, the recycling center can also accept a certain degree of profit concession in order to maintain the stability and coordination of the supply chain system. 

(2) In the process of introducing online channels into the recycling center, when fairness concerns occur due to the high proportion of offline channels, it will also urge the recycling center to re-determine the channel pricing with the TPR by means of channel power and bargaining. This behavior of the recycling center led to a significant reduction in the transfer price and the recycling price in a DRSC’s offline channels, and a small reduction in the recycling price in online channels. Although this price adjustment leads to an increase in online channel recovery compared with offline channels, it results in a decrease in TPR, supply chain system, and its own revenue. This behavior of the recycling center will definitely damage the TPR’s recycling enthusiasm in the long run and cause losses to the entire recycling industry. It can be said that this behavior of the recycling center is “harming others and harming oneself”. 

(3) Whether it is a recycling center or a TPR, when its fairness concern behavior is at different levels, its impact on enterprise pricing and profit is also different. It can be clearly found that when the fairness concern factor is at a lower level, its unit change has a significantly higher impact on pricing and profits than when it is at a higher level. Therefore, for business decision-makers, concerns about fair behavior should be kept at an appropriate level. Because although when at the primary level, fairness concern behavior can satisfy the enterprise’s sense of fairness and has a certain impact on the profits of other enterprises, but excessive concerns for fairness behavior has little impact on the profits of other enterprises. Even this excessive concern may lead to conflicts among supply chain members, seriously damaging the interests of all parties and ultimately affecting the long-term development in the future. 

We believe that no matter for the benefit of itself, other supply chain members or the whole supply chain system, the recycling center and TPR should always control the level of fairness concerns of themselves and other supply chain members, promote the coordination of the supply chain, and at the same time do not do things such as retaliatory pricing that damage the profits of the supply chain system. Specifically, on the one hand, the recycling center can reduce the TPR’s unfairness in the supply chain by giving the TPR a certain degree of profit sharing, price discounts or technical guidance. On the other hand, the recycling center also needs to continuously communicate with the TPR to promote mutual understanding and coordination of the supply chain. In addition, the TPR needs to consider the benefits of the recycling center and the supply chain system while reasonably striving for its own benefits, and constantly strengthen communication and learning with the recycling center in the aspects of the recycling process and technology. 

### 5.2. Future Directions

In this subsection, we discussed the shortcomings of this research and some directions that can be expanded in the future. On the one hand, we studied a DRSC structure composed of a recycling center and TPR, but in fact, a DRSC structure composed of a recycling center, TPR, and third-party recycling platform has appeared in China so far. The third-party recycling platform is a partner to the recycling center and a competitor to a TPR. Therefore, it is meaningful to study and consider the DRSC problem of the third-party recycling platform. On the other hand, the information assumed in this study is complete and accurate, and there is no information asymmetry or information distortion. However, in reality, there may be situations where information recipients or publishers exaggerate or weaken information content for some purpose, or information asymmetry and distortion may result from different reasons such as attitude, experience, and expectation. This problem can also be used as the next research direction.

## Figures and Tables

**Table 1:** Notation.

Symbol	Definition
*d_r_*	The amount of WEEE recovered from offline channels.
*d_e_*	The amount of WEEE recovered from online channels.
*θ*	Proportion of consumers who prefer online channels (0 < *ϴ* < 1).
*p*_0_	Revenue from disposal of WEEE by recycling centers.
*p_t_*	The TPR’s price per unit WEEE recovered from consumers.
*p_e_*	The price of WEEE unit that the recycling center recycles from consumers.
*w*	Transfer price of WEEE unit recovered from the TPR by the recycling center.
*c*	Cost of recycling on the online channel.
*a*	The amount of basic recovery in the market that is not affected by the recovery price (*a* > 0).
*β*	The elasticity coefficient of the change in the recycling amount of this channel caused by the change in the recycling price of its own channel unit (*β* > 0).
*κ*	The elasticity coefficient of the change of self-recovery quantity caused by the change of the recovery price of the opposite channel unit (*β* > *κ* > 0).
*λ*	The fairness concern coefficient of the TPR (*λ* > 0).
*η*	The fairness concern coefficient of the recycling center (*η* > 0).
∏_*m*_	The benefits of the recycling center.
∏_*t*_	The benefits of the TPR.
∏	The total revenue of the supply chain system is *∏_m_ + ∏_t_*.
△∏	The profit difference between the recycling center and TPR is *∏_m_* − *∏_t_*.
*μ_m_*	Fair utility function of the recycling center.
*μ_t_*	Fair utility function of the TPR.

WEEE refers to waste electrical and electronic equipment, while TPR refers to third party recycler.

**Table 2:** The effect of *λ* on decisions and profits.

*λ*	*P_e_*	*P_t_*	*W*	*∏_m_*	*∏_t_*	*∏*	*△∏*
0	66.9	60.1	88.1	35591.3	3920.0	39511.3	31671.3
0.1	66.9	60.1	92.8	34937.9	4573.3	39511.3	30364.6
0.2	66.9	60.1	96.1	34471.3	5040.0	39511.3	29431.3
0.3	66.9	60.1	98.6	34121.3	5390.0	39511.3	28731.3
0.4	66.9	60.1	100.6	33849.0	5662.2	39511.3	28186.8
0.5	66.9	60.1	102.1	33631.3	5880.0	39511.3	27751.3
0.6	66.9	60.1	103.4	33453.1	6058.2	39511.3	27394.9
0.7	66.9	60.1	104.5	33304.6	6206.7	39511.3	27097.9
0.8	66.9	60.1	105.4	33178.9	6332.3	39511.3	26846.6
0.9	66.9	60.1	106.1	33071.3	6440.0	39511.3	26631.3
1	66.9	60.1	106.8	32977.9	6533.3	39511.3	26444.6

**Table 3:** The effect of *η* on decisions and profits.

*η*	*P_t_*	*W*	*P_e_*	*∏_t_*	*∏_m_*	*∏*	*△∏*
0.0	60.1	88.1	66.9	3920.0	35591.3	39511.3	31671.3
0.1	58.9	85.7	66.9	3586.5	35576.4	39163.0	31989.9
0.2	58.0	83.8	66.9	3340.1	35544.9	38885.0	32204.7
0.3	57.2	82.3	66.9	3150.9	35507.3	38658.3	32356.4
0.4	56.6	81.1	66.9	3001.3	35468.8	38470.0	32467.5
0.5	56.1	80.1	66.9	2880.0	35431.3	38311.3	32551.3
0.6	55.7	79.3	66.9	2779.8	35395.8	38175.6	32616.0
0.7	55.3	78.6	66.9	2695.7	35362.7	38058.4	32667.0
0.8	55.0	77.9	66.9	2624.1	35332.1	37956.2	32707.9
0.9	54.8	77.4	66.9	2562.5	35303.8	37866.2	32741.3
1.0	54.5	76.9	66.9	2508.8	35277.7	37786.5	32768.9

## Appendix A

**Proof** **of** **Property** **1.** The Hessian matrix of ∏_*m*_(*p_e_*, *w*) is [∂2∏m∂pe2∂2∏m∂pe∂w∂2∏m∂w∂pe∂2∏m∂w2], and after simplifying it, we can get $\left[\begin{array}{cc} -\frac{41}{5} & 3\\ 3 & -5 \end{array}\right]$. Since the order principal minor determinant of the matrix is $-\frac{41}{5}≺0$, $-\frac{41}{5}*\left(-5\right)-3*3=32≻0$. Therefore, the matrix is a negative definite matrix. ∏_*m*_(*p_e_*, *w*) is strictly concave and has a maximum value. □

**Proof** **of** **Property** **2.** The Hessian matrix of $\prod_{m}(p_{e},w)$ is [∂2∏m∂pe2∂2∏m∂pe∂w∂2∏m∂w∂pe∂2∏m∂w2], and after simplifying it, we can get $\left[\begin{array}{cc} -10+\frac{18}{10+10\lambda } & 3-\frac{3}{2+2\lambda }+\frac{3(5+10\lambda )}{10+10\lambda }\\ 3-\frac{3}{2+2\lambda }+\frac{3(5+10\lambda )}{10+10\lambda } & -2\frac{2(5+10\lambda )}{2+2\lambda } \end{array}\right]$. Since the order principal minor determinant of the matrix is $-10+\frac{18}{10+10\lambda }≺0$, $73+138\lambda -16\lambda^{2}≻0$. Therefore, the matrix is a negative definite matrix. ∏_*m*_(*p_e_*,*w*) is strictly concave and has a maximum value. □

**Proof** **of** **Property** **3.** The Hessian matrix of *μ_m_*(*p_e_*, *w*) is [∂2μm∂pe2∂2μm∂pe∂w∂2μm∂w∂pe∂2μm∂w2], and after simplifying it, we can get $\left[\begin{array}{cc} -\frac{9\eta }{10}-\frac{41(1+\eta )}{5} & \frac{3\eta }{2}+3(1+\eta )\\ \frac{3\eta }{2}+3(1+\eta ) & -\frac{5\eta }{2}-5(1+\eta ) \end{array}\right]$. Since the order principal minor determinant of the matrix is $-\frac{9\eta }{10}-\frac{41(1+\eta )}{5}≺0$, $269\eta^{2}+966\eta +784≻0$. Therefore, the matrix is a negative definite matrix. $\mu_{m}(p_{e},b)$ is strictly concave and has maximum value. □
